# Triptolide Inhibits Preformed Fibril-Induced Microglial Activation by Targeting the MicroRNA155-5p/SHIP1 Pathway

**DOI:** 10.1155/2019/6527638

**Published:** 2019-04-28

**Authors:** Yang Feng, Chuyun Zheng, Yajun Zhang, Changyang Xing, Wenbin Cai, Ruru Li, Jianzong Chen, Yunyou Duan

**Affiliations:** ^1^Department of Ultrasound Diagnosis, Tangdu Hospital, Fourth Military Medical University, Xi'an 710038, China; ^2^Department of Traditional Chinese Medicine, Xijing Hospital, Fourth Military Medical University, Xi'an 710038, China; ^3^Department of Ultrasound Diagnosis, Xi'an Chest Hospital, Xi'an 710100, China; ^4^Department of Ultrasound Diagnostics, Beijing Tongren Hospital, Capital Medical University, Beijing 100730, China

## Abstract

Evidence suggests that various forms of *α*-synuclein- (*α*Syn-) mediated microglial activation are associated with the progression of Parkinson's disease. MicroRNA-155-5p (miR155-5p) is one of the most important microRNAs and enables a robust inflammatory response. Triptolide (T10) is a natural anti-inflammatory component, isolated from a traditional Chinese herb. The objective of the current study was to identify the role and potential regulatory mechanism of T10 in *α*Syn-induced microglial activation via the miR155-5p mediated SHIP1 signaling pathway. Mouse primary microglia were exposed to monomers, oligomers, and preformed fibrils (PFFs) of human wild-type *α*Syn, respectively. The expressions of TNF*α* and IL-1*β*, measured by enzyme-linked immunosorbent assay (ELISA) and qPCR, demonstrated that PFFs initiated the strongest immunogenicity in microglia. Application of inhibitors of toll-like receptor (TLR) 1/2, TLR4, and TLR9 indicated that PFFs activated microglia mainly via the NF-*κ*B pathway by binding TLR1/2 and TLR4. Treatment with T10 significantly suppressed PFF-induced microglial activation and attenuated the release of proinflammatory cytokines including TNF*α* and IL-1*β*. Levels of IRAK1, TRAF6, IKK*α*/*β*, p-IKK*α*/*β*, NF-*κ*B, p-NF-*κ*B, PI3K, p-PI3K, t-Akt, p-Akt and SHIP1 were measured via Western blot. Levels of miR155-5p were measured by qPCR. The results demonstrated that SHIP1 acted as a downstream target molecule of miR155-5p. Treatment with T10 did not alter the expression of IRAK1 and TRAF6, but significantly decreased the expression of miR155-5p, resulting in upregulation of SHIP1 and repression of NF-*κ*B activity, suggesting inhibition of inflammation and microglial activation. The protective effects of T10 were abolished by the use of SHIP1 siRNA and its inhibitor, 3AC, and miR155-5p mimics. In conclusion, our results demonstrated that treatment with T10 suppressed microglial activation and attenuated the release of proinflammatory cytokines by suppressing NF-*κ*B activity via targeting the miR155-5p/SHIP1 pathway in PFFs-induced microglial activation.

## 1. Introduction

Parkinson's disease (PD) is the second most common neurodegenerative disorder and is characterized by a progressive deterioration of vulnerable dopaminergic neurons and the presence of intraneuronal aggregates known as Lewy bodies composed of *α*-synuclein (*α*Syn) [1]. Recent studies have identified several non-cell-autonomous mechanisms of PD, including microglial activation and proinflammatory cytokine release [2]. More specifically, various forms of *α*Syn, including monomers, oligomers, and preformed fibrils (PFFs) derived from apoptotic dopaminergic neurons are potential endogenous antigens, capable of activating microglia and stimulating the release of more proinflammatory cytokines [3, 4]. This results in positive feedback leading to further apoptosis of dopaminergic neurons, which is an important pathogenesis of PD. Among these, PFFs show pathogenicity of prion-like spreading in the brain with seeding capacity, which initiate the strongest immunogenicity in microglia [5]. Thus, identification of novel therapeutic agents and methods against the PFF-induced microglial activation is important.

It is evident that microRNAs (miR), which are highly conserved small noncoding RNAs, are pivotal positive and negative regulators of inflammatory response [6]. These participate in various regulatory network motifs. Specifically, miR-155-5p, which regulates macrophage inflammatory response by forming a positive regulatory loop that alters NF-*κ*B activity, has been extensively characterized [7, 8]. However, the biological functions of miR155-5p need further exploration, particularly in relation to PD.

Triptolide (T10) is a monomeric component isolated from a Chinese herb, *Tripterygium wilfordii Hook F*, which is characterized by anti-inflammatory and anticancer activity [9, 10]. Additionally, it suppresses disturbances produced by cell-autonomous mechanisms in PD, such as aggregation of *α*Syn and disruption of the autophagy-lysosome system [11]. However, at present, few studies have determined the effect of T10 on the miRNAome related to *α*Syn-induced microglial activation in PD.

The current study investigated the mechanism of PFF-induced microglial activation and the functional interaction between T10, miR155-5p, and NF-*κ*B activity during the stimuli produced by PFFs in primary microglia.

## 2. Method

### 2.1. Reagents

T10 and human wild-type *α*Syn were purchased from Sigma-Aldrich (St. Louis, MO, USA). 4-Hydroxynonenal (HNE) was purchased from Abcam (USA), while miR155-5p mimics, inhibitor, and negative control were purchased from Rio Bio (Guangzhou, China). The pRL-TK plasmids were purchased from GenePharma (Shanghai, China). SHIP1 siRNA and scrambled control siRNA were purchased from GeneChem (Shanghai, China). Lipofectamine 2000 and Lipofectamine RNAiMAX were purchased from Invitrogen (Carlsbad, CA, USA). TNF*α* and IL-1*β* enzyme-linked immunosorbent assay kits were purchased from Abcam (USA). Cu-CPT22, resatorvid, E6446, and 3*α*-aminocholestane (3AC) were purchased from MedchemExpress (New Jersey, USA). Antibodies against IRAK1, TRAF6, p-IKK*α*/*β*, IKK*α*/*β*, NF-*κ*B (p65), p-NF-*κ*B (phospho-p65), PI3K (p85), p-PI3K (phosphor-p85), t-Akt, p-Akt, SHIP1, and *β*-actin were purchased from Cell Signaling Technology (Beverly, MA, USA). Goat anti-rabbit and goat anti-mouse secondary antibodies were purchased from Boster (Wuhan, China). Alexa Fluor 568 anti-rabbit secondary antibody and DAPI were purchased from Invitrogen (Carlsbad, CA, USA). Luciferase Reporter Gene Assay kit was purchased from Roche (Basel, Switzerland).

### 2.2. Preparation of Recombinant *α*Syn Species

Monomeric *α*Syn was purchased from Sigma (USA). In order to construct oligomers, the protein was dissolved in PBS, generating a concentration of 1 mg/ml and incubated with HNE (Abcam, USA) at 37°C for 24 h with a *α*Syn : HNE M at a ratio of 30 : 1. Following incubation, residual aldehyde was removed with an Amicon 3 kDa cut-off ultra-centrifugal unit (Millipore, USA) at 4000×g for 40 min (3-16KL, Sigma, Germany). *α*Syn oligomerization was determined by SEC-HPLC analysis, as previously described [12].

PFFs were produced via agitation of 5 mg/ml protein in a ThermoMixer (Eppendorf, USA) for 7 days at 1000 rpm in a 37°C incubator. Thioflavine T (ThT) assay was performed to confirm successful formation of fibrils.

### 2.3. Primary Culture of Microglia and Treatments

Microglia were prepared from cerebral cortices of 1-3-day-old neonatal C57BL/6 mice as previously described [13]. Briefly, a cell suspension of dissociated brain tissues was prepared. Next, the isolated cells were cultured for 2 weeks in Dulbecco's modified Eagle's medium/F12 (Gibco, USA) containing 10% fetal bovine serum with 1% penicillin and streptomycin. The microglia were purified in flasks shaken at 180 rpm, 37°C for 3 h (SI-200, Major Science, USA). OX-42 and Iba-1 staining was used to determine the purity of enriched microglia (data not shown). A purity of 95% was considered as satisfactory.

PFF was sonicated with 60 pulses at 10% power (total of 30 s at 0.5 s on and 0.5 s off) before use. Primary microglia were treated with 2 *μ*M PFFs for 12 h and with or without 40 nM T10 (Sigma-Aldrich, USA) for a further 12 h. Also, 2 *μ*M monomers and oligomers were allowed to form in cultured cells for the purpose of comparing immunogenicity. Equivalent PBS and 0.1 *μ*g/ml LPS were added as negative and positive controls, respectively.

### 2.4. Inhibitors

The final working concentration of TLR1/2 receptor inhibitor Cu-CPT22, TLR4 receptor inhibitor resatorvid, TLR9 receptor inhibitor E6446, and SHIP1 inhibitor 3AC were 10 *μ*M, 10 *μ*M, 50 nM, and 20 *μ*M, respectively. Cells were pretreated with inhibitors for 12 h and then subjected to further treatments. All inhibitor and DMSO control-group concentrations were optimal as per our previous experiments (data not shown). All inhibitors were purchased from MedchemExpress (New Jersey, USA).

### 2.5. siRNA and Plasmid Transfection

miR155-5p mimics, miR155-5p inhibitors, and the controls (RiboBio, China) were transfected into primary microglia using Lipofectamine 2000 (Invitrogen, USA). SHIP1 siRNA or recommended scrambled siRNA (GeneChem, China) was transfected using Lipofectamine RNAiMAX (Invitrogen, USA) according to the manufacturer's protocol.

### 2.6. ELISA

Cytokine levels were determined in the supernatants collected from primary microglia. TNF*α* and IL-1*β* enzyme-linked immunosorbent assays (Abcam, USA) were performed according to the manufacturer's protocol. Absorbance was measured at 450 nm. The resulting concentrations were adjusted to final concentrations according to dilution folds.

### 2.7. qRT-PCR

Total RNA were extracted using TRIzol reagent (Invitrogen, USA) and reverse-transcribed using the TaqMan Universal Master Mix (Applied Biosystems, USA) or PrimeScript™ RT reagent Kit (TaKaRa, Japan) according to the manufacturer's protocol. The primers used in this study were obtained from Applied Biosystems. The qRT-PCR reaction with SYBR Green (TaKaRa, Japan) was performed using a Real-time PCR Detection System (CFX96, Bio-Rad, USA) in the 25 *μ*l reaction mixtures. The expression levels of the target genes were analyzed using the 2^−ΔΔCt^ method.

### 2.8. Western Blot

Following sonication and centrifugation, the supernatants were diluted with 5x SDS loading buffer and separated via SDS-PAGE. Next, the proteins were transferred to PVDF membranes (Millipore, USA). After blocking for 2 h, with 5% skim milk in Tris-buffered saline at 37°C, the membrane was probed with primary antibodies against IRAK1, TRAF6, p-IKK*α*/*β*, IKK*α*/*β*, NF-*κ*B, p-NF-*κ*B, PI3K, p-PI3K, t-Akt, p-Akt, SHIP1, and *β*-actin overnight at 4°C. Next, the membranes were incubated with appropriate secondary antibodies for 1 h at 37°C. Bands visualized using a Bio-Rad imaging system (Bio-Rad, USA) were analyzed using ImageJ software.

### 2.9. Immunofluorescence

Cells were fixed with 4% paraformaldehyde for 1 h followed by permeabilization in 0.3% Triton X-100 for 30 min. Next, cells were blocked with 5% bovine serum albumin for 1 h and incubated with primary antibody against NF-*κ*B overnight at 4°C. After washing with PBS, the cells were incubated with Alexa Fluor 568-conjugated secondary antibody for 2 h at room temperature. The nuclei were then stained with DAPI for 8 min. Images were acquired via confocal microscopy (A1R, Nikon, Japan).

### 2.10. Luciferase Reporter Assay

The pRL-TK plasmid containing a Renilla luciferase gene for internal normalization and various constructs containing pMIR-SHIP1 and pMIR-SHIP1-mut were purchased from GenePharma. HEK-293T cells were seeded into 96-well plates and transfected with plasmids by using Lipofectamine 2000. Following incubation for 24 h, cells were harvested and luciferase activity was measured using the Luciferase Reporter Gene Assay kit (Roche, Switzerland) according to the manufacturer's protocol.

### 2.11. Statistics

GraphPad software, version 7, was used to analyze the data which are presented as mean ± standard error of the mean (SEM). One-way ANOVA test was applied to assess statistical significance, which was set at *p* < 0.05.

## 3. Results

### 3.1. PFFs Induce the Strongest Immunogenicity in Microglia

**Figure 1:**
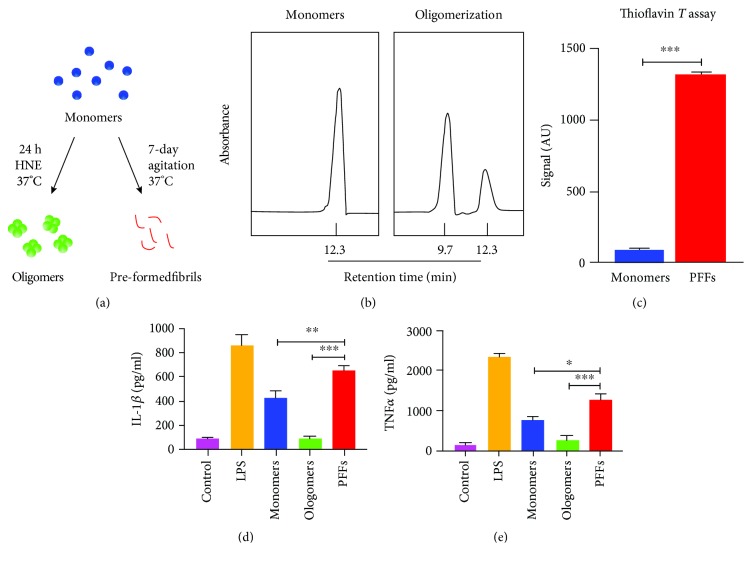
*α*Syn activates primary microglial aggregation-dependently. (a) Schematic overview of the preparation of different *α*Syn species. (b) Size exclusion chromatography HLPC (SEC-HLPC) was performed to determine the HNE-mediated oligomerization after being incubated at 37°C for 24 h. (c) Thioflavin T was used to confirm the beta sheet conformation that characterizes the amyloid-like PFFs after agitation for 7 days at 37°C. Levels of TNF*α* (d) and IL-1*β* (e) were measured after exposing to monomers, oligomers, or PFFs for 24 h in primary microglia. The values were expressed as mean ± SEM. ^∗^ *p* < 0.05, ^∗∗^ *p* < 0.005, and ^∗∗∗^ *p* < 0.001.

Prior to stimulating primary microglia with *α*Syn, physicochemical properties of oligomers and PFFs were identified through SEC-HPLC and ThT assay, respectively (Figures 1(b) and 1(c)). The effects of different aggregations of *α*Syn (monomers, oligomers, and PFFs) on the inflammatory response were evaluated. Exposure of PFFs induced a profound release of TNF*α* and IL-1*β*. Stimulation with monomers also induced a similar release of TNF*α* and IL-1*β*; however, the amounts were far lower compared to that with PFF stimulation (Figures 1(d) and 1(e)). Notably, oligomers appeared to be immunogen-free towards primary microglia, and changes were not observed following treatment with oligomers.

### 3.2. PFFs Activate NF-*κ*B Signaling by Both TLR1/2 and TLR4

**Figure 2:**
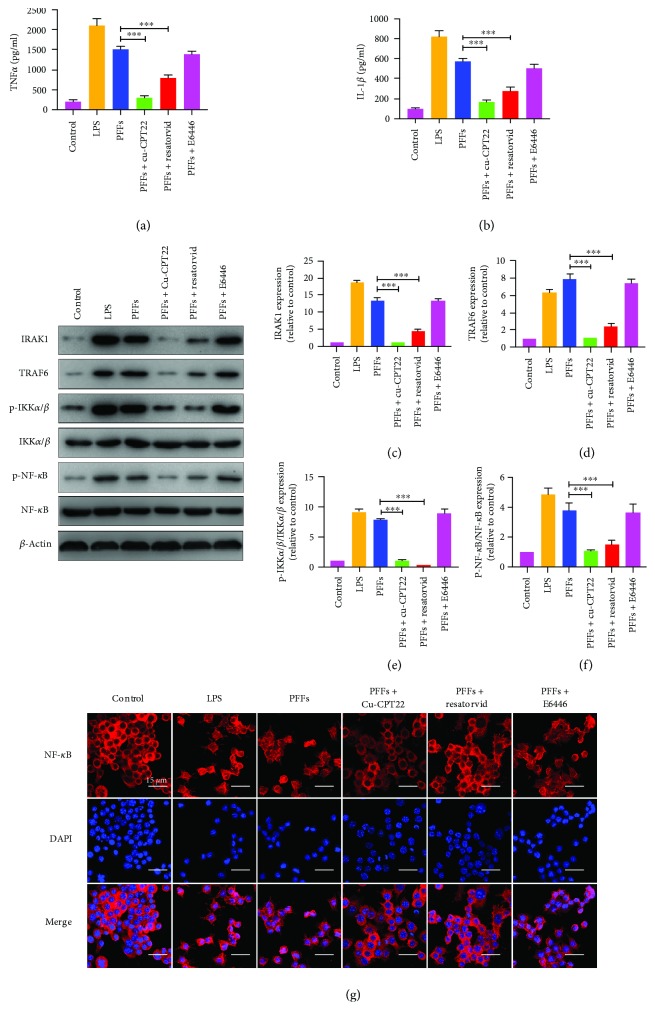
PFFs activated primary microglia by binding to TLR1/2 or TLR4. Primary microglia were pretreated with Cu-CPT22, resatorvid, or E6446 for 12 h and then treated with PFFs for further 24 h. Levels of TNF*α* (a) and IL-1*β* (b) were measured by ELISA assay. (c-f) Expressions of IRAK1, TRAF6, p-IKK*α*/*β*/IKK*α*/*β* ratio, and p-NF-*κ*B/NF-*κ*B ratio were measured by Western blot. (g) Translocations of NF-*κ*B were monitored by immunofluorescence staining. Scale bar = 15 *μ*m. The values were expressed as mean ± SEM. ^∗∗∗^ *p* < 0.001.

In order to determine the involvement of TLRs in PFF-induced primary microglial activation, highly selective inhibitors of TLR1/2, TLR4, and TLR9 were applied to avoid cross-inhibition [14–16]. PFF-induced release of TNF*α* and IL-1*β* was completely abolished by Cu-CPT22 and partially abolished by resatorvid, but not by E6446 (Figures 2(a) and 2(b)). Expressions of IRAK1 and TRAF6, two crucial adaptors for TLR-mediated NF-*κ*B signaling, were measured. Cu-CPT22 suppressed IRAK1 and TRAF6 expression as well as phosphorylation of IKK*α*/*β* and NF-*κ*B, induced by PFFs. Resatorvid demonstrated effects similar to that of Cu-CPT22, but at a lesser level. E6446 did not show significant inhibitory effects on PFF-induced inflammation (Figures 2(c)–2(f)). Immunofluorescent results indicated that translocation of NF-*κ*B induced by PFFs may be fully prevented by Cu-CPT22 and partially blocked by resatorvid, while E6446 did not have any effects (Figure 2(g)).

### 3.3. PI3K/Akt Pathway Was Involved in the Repression of T10 on the Expression of miR155-5p and the NF-*κ*B Activity in Primary Microglia

**Figure 3:**
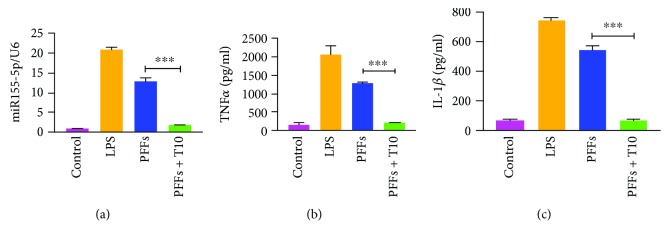
T10 treatment suppressed expression of miR155-5p and microglial activation. Primary microglia were treated with PFFs for 12 h and then treated with or without T10 for additional 12 h. (a) qRT-PCR results of miR155-5p. Levels of TNF*α* (b) and IL-1*β* (c). The values were expressed as mean ± SEM. ^∗∗∗^ *p* < 0.001.

T10 significantly prevented elevation of miR155-5p and reduced the release of TNF*α* and IL-1*β* induced by PFFs, leading to the downregulated inflammatory status of microglia (Figures 3(a)–3(c)).

**Figure 4:**
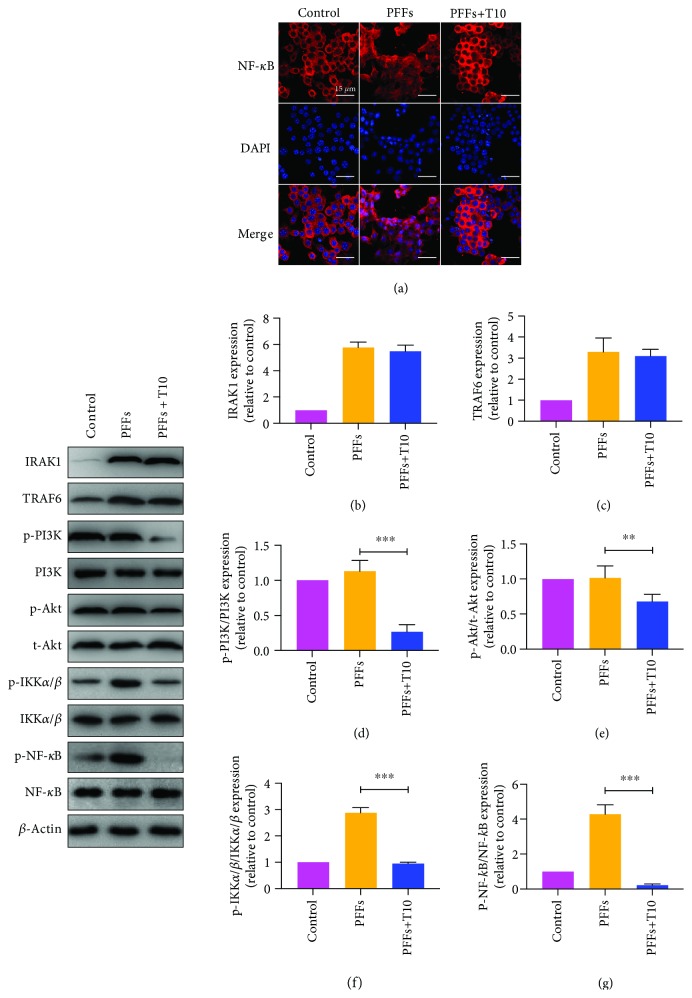
T10 suppressed NF-*κ*B activity via the PI3K/Akt pathway. (a) Immunofluorescence staining of NF-*κ*B. Scale bar = 15 *μ*m. (b-g) Treatment of T10 did not affect the expressions of IRAK1 and TRAF6, but could downregulate phosphorylation of PI3K, Akt, IKK*α*/*β*, and NF-*κ*B. The values were expressed as mean ± SEM. ^∗∗^ *p* < 0.005 and ^∗∗∗^ *p* < 0.001.

We further examined the effects of T10 on the NF-*κ*B pathway. Immunofluorescence staining of NF-*κ*B showed that T10 significantly decreased the translocation into the nucleus (Figure 4(a)). Interestingly, although p-IKK*α*/*β* and p-NF-*κ*B levels were significantly downregulated after treatment of T10, primary microglia did not show any changes in IRAK1 or TRAF6 levels, indicating that T10 may influence the NF-*κ*B pathway in other interventions (Figures 4(b)–4(g)). According to previous studies, besides IRAK1/TRAF6, PI3K/Akt is also an intracellular pathway vital for regulating IKK*α*/*β* phosphorylation [17]. To determine whether the PI3K/Akt pathway is involved in the repression of T10 on NF-*κ*B activity, the phosphorylation of PI3K and Akt was assessed by Western blot. The results showed that treatment of T10 significantly decreased the expression of p-PI3K and p-Akt. Interestingly, treatment of PFFs did not alter the expression of p-PI3K or p-Akt, suggesting that T10 and PFFs may regulate NF-*κ*B activity through two independent pathways and that IKK*α*/*β* may be a cross-molecular component of the two pathways.

### 3.4. Downregulation of miR155-5p Alleviates PFF-Induced Inflammatory Response

**Figure 5:**
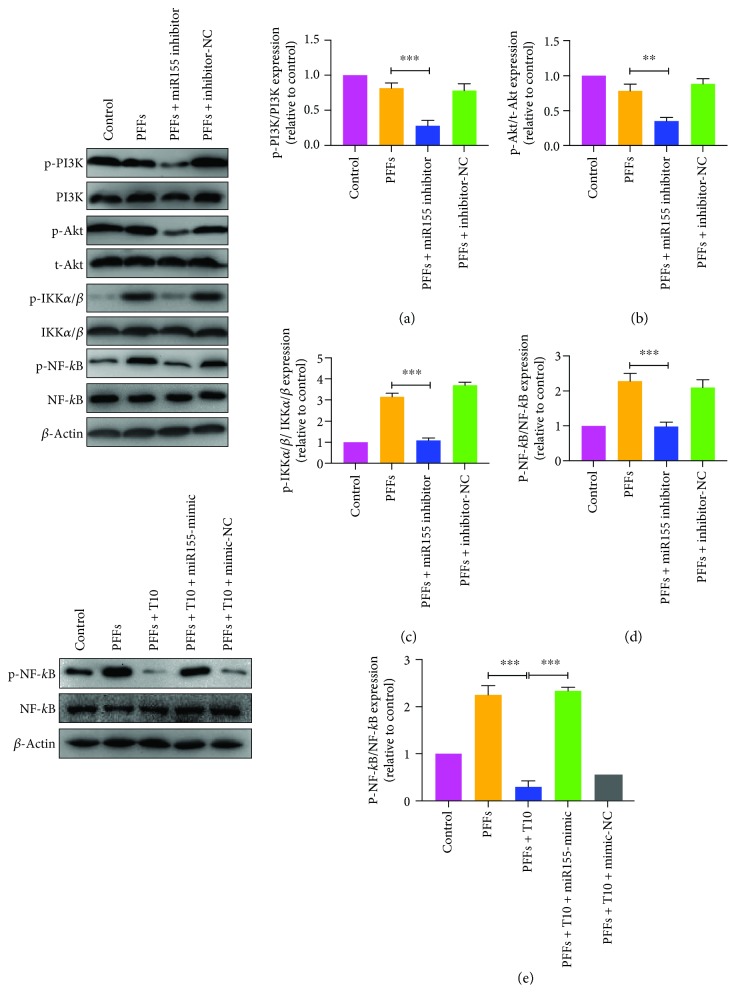
miR155-5p regulated NF-*κ*B activity by the PI3K/Akt pathway. (a-d) Primary microglia were cotreated with miR155-5p inhibitors and PFFs for 24 h and harvested for analysis. Ratios of p-PI3K/PI3K, p-Akt/t-Akt, p-IKK*α*/*β*/IKK*α*/*β*, and p-NF-*κ*B/NF-*κ*B were measured by Western blot, and representative images are shown. (e) Phosphorylation of NF-*κ*B was evaluated after treatment of miR155-5p mimics. The values were expressed as mean ± SEM. ^∗∗^ *p* < 0.005 and ^∗∗∗^ *p* < 0.001.

Considering the increase of miR155-5p following PPF treatment as well as its decrease following T10 treatment, we investigated the potential role of miR155-5p in NF-*κ*B activity by altering miR155-5p levels with its inhibitors and mimics. Treatment with miR155-5p inhibitors suppressed phosphorylation of PI3K, Akt, and IKK*α*/*β*, as well as the expression of p-NF-*κ*B (Figures 5(a)–5(d)). This resembled the effect of T10 treatment. On the contrary, treatment with miR155-5p mimics reversed the repression of T10 on NF-*κ*B activity in PFF-treated primary microglia (Figure 5(e)).

### 3.5. SHIP1 Is Requisite for the Regulatory Effect of miR155-5p on NF-*κ*B in Primary Microglia

**Figure 6:**
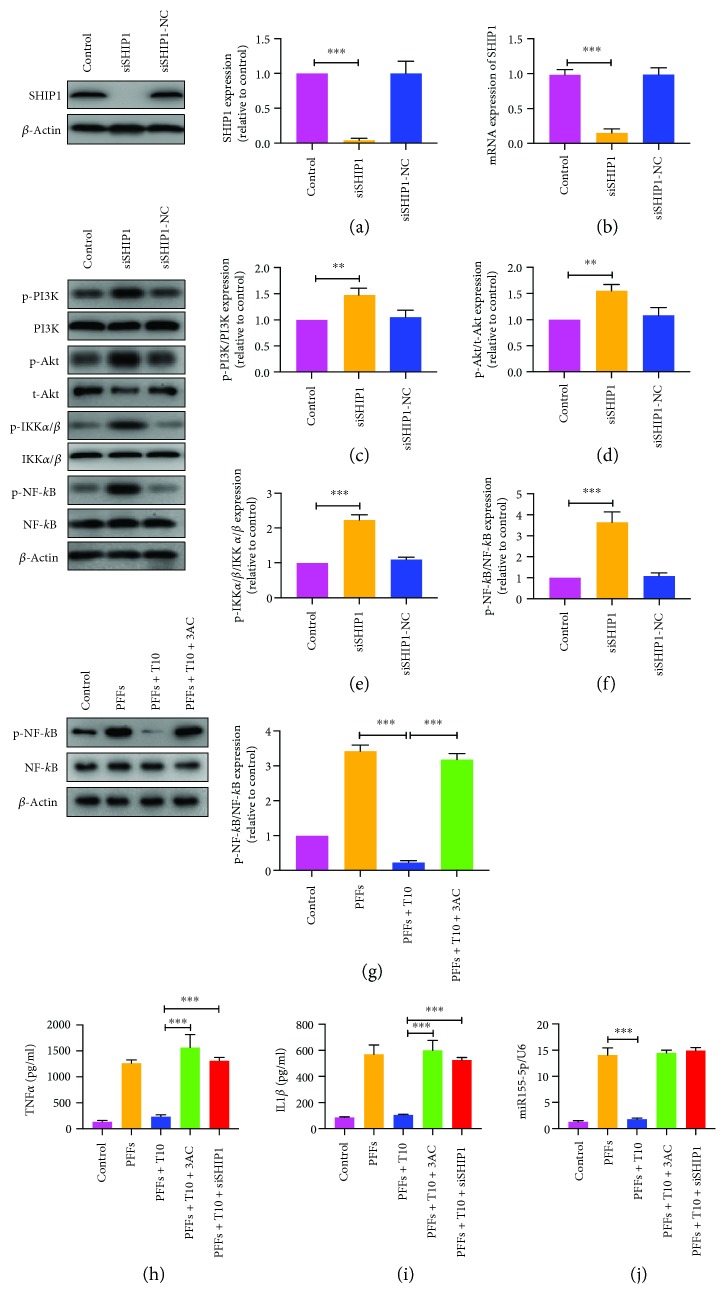
SHIP1 was involved in the regulation of T10 on NF-*κ*B activity. The mRNA (a) and protein (b) levels of SHIP1 in primary microglia transfected with SHIP1 siRNA. (c-f) Ratios of p-PI3K/PI3K, p-Akt/t-Akt, p-IKK*α*/*β*/IKK*α*/*β*, and p-NF-*κ*B/NF-*κ*B were measured by Western blot in SHIP1-silenced primary microglia. (g) SHIP1 inhibitor, 3AC, abolished the inhibition of T10 on the ratio of p-NF-*κ*B/NF-*κ*B. (h-i) Levels of TNF*α* and IL-1*β*. (j) Levels of miRNA155-5p. The values were expressed as mean ± SEM. ^∗∗^ *p* < 0.005 and ^∗∗∗^ *p* < 0.001.

**Figure 7:**
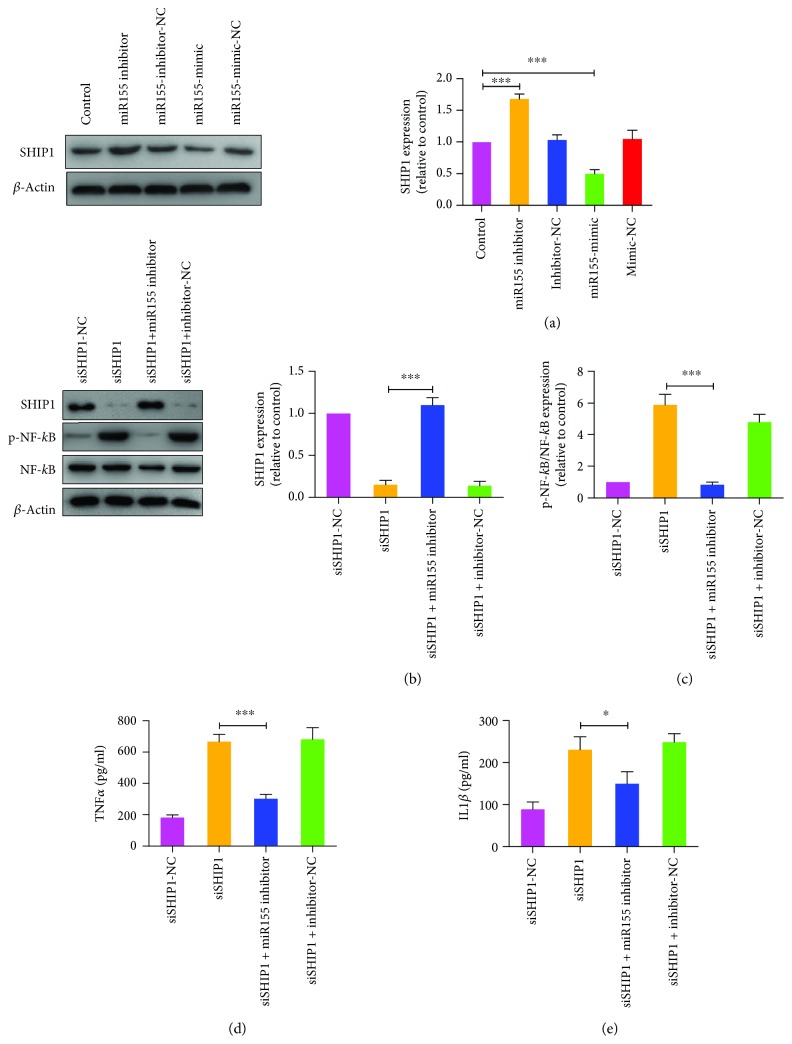
The role of SHIP1 in the release of TNF*α* and IL-1*β* inhibited by miR155-5p in primary microglia. (a) Expression of SHIP1 after treatment of miR155-5p mimics or inhibitors in primary microglia. (b-e) Levels of SHIP1, TNF*α*, and IL-1*β* after treatment of miR155-5p inhibitors in SHIP1 silenced primary microglia. The values were expressed as mean ± SEM. ^∗^ *p* < 0.05 and ^∗∗∗^ *p* < 0.001.

**Figure 8:**
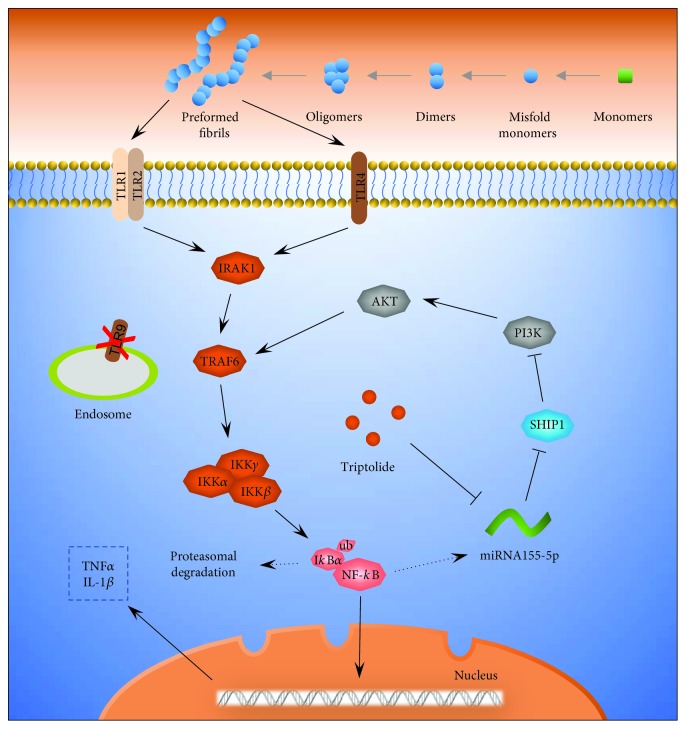
T10 inhibited NF-*κ*B activity via miR155-5p/SHIP1 axis. PPFs may bind to the TLR1/2 or TLR4; IKK*α*/*β* is therefore activated through IRAK1/TRAF6 signaling. IKK*α*/*β*, in turn, phosphorylated I*κ*B*α*, which results in dissociation of the I*κ*B*α* form NF-*κ*B. Activated NF-*κ*B is then translocated into nuclei where it binds to specific sequences of DNA, resulting in upregulation of TNF*α* and IL-1*β*. T10 could inhibit the expression of miR155-5p, which is capable of negatively regulating SHIP1 expression. Overexpressed SHIP1 mediated by T10, in turn, inhibits activation of IKK*α*/*β* through PI3K/Akt signaling, finally resulting in inhibition of NF-*κ*B activity and corresponding repression of TNF*α* and IL-1*β*.

According to TargetScan search results, SHIP1 is a predictive target for miR155-5p (Supplementary Figure 1a). We verified whether SHIP1 expression was hampered by hybridization between SHIP1 3′-UTR and miR155-5p via the luciferase report assay. The results indicated that transfection with miR155-5p mimics significantly repressed luciferase activity in wild-type SHIP1 3′-UTR, while the mutant did not (Supplementary Figure 1b). Therefore, we investigated the effect of SHIP1 on miR155-5p function in primary microglia. Upon transfecting primary microglia with SHIP1 siRNA, the endogenous mRNA and SHIP1 protein levels were significantly decreased, suggesting that the siRNA functioned perfectly (Figures 6(a) and 6(b)). Furthermore, SHIP1 knockdown significantly increased the expressions of p-PI3K, p-Akt, p-IKK*α*/*β*, and p-NF-*κ*B, suggesting aggravation of inflammation (Figures 6(c)–6(f)). Treatment with SHIP1 Inhibitor, 3AC, also promoted p-NF-*κ*B expression, which was similar to the effect of SHIP1 siRNA (Figure 6(g)). Both SHIP1 siRNA and 3AC inhibited negative regulation of TNF*α* and IL-1*β* release by T10, without altering miR155-5p expression (Figure 6(h)–6(j)). Additionally, changes in the miR155-5p levels caused significant fluctuations in SHIP1 expression, indicating that miR155-5p may negatively regulate the expression of SHIP1 (Figure 7(a)). The results of Western blot indicated that when SHIP1-silenced cells were treated with miR155-5p inhibitors, SHIP1 expression was elevated and p-NF-*κ*B expression was decreased (Figures 7(b) and 7(c)). Furthermore, miR155-5p inhibitors reversed the enhancement effect of SHIP1 siRNA on the expression of TNF*α* and IL-1*β* (Figures 7(d) and 7(e)). These results indicated that T10 may suppress the NF-*κ*B activity and reduced release of proinflammatory cytokines potentially through the miR155-5p/SHIP1 pathway. Taken together, our findings indicate that PFFs activate NF-*κ*B signaling through binding TLR1/2 or TLR4 and T10 is capable of suppressing NF-*κ*B activation induced by PFFs through the miR155-5p/SHIP1 pathway (Figure 8).

## 4. Discussion

The current study investigated a PFF-based neuroinflammation model in order to analyze the anti-inflammatory effects of T10 on PFF-induced neuroinflammation. Our findings demonstrated that PFFs induced neuroinflammation via activation of NF-*κ*B signaling by binding either TLR1/2 or TLR4 and that T10 suppressed NF-*κ*B activation and reduced the release of proinflammatory cytokines in primary microglia. Moreover, the miR155-5p/SHIP1 pathway is critical for the regulation of NF-*κ*B activity during PFF-induced neuroinflammation. The mechanism underlying the anti-inflammation role of T10 may be inhibition of miR155-5p expression and the subsequent upregulation of SHIP1, leading to the repression of NF-*κ*B activity.

PD is characterized by a slow and progressive degeneration of dopaminergic neurons in the substantia nigra and the presence of proteinaceous inclusions known as Lewy bodies, which are composed of *α*Syn [18]. Despite intensive studies, the cause of neuronal loss in PD and the presence of protein inclusions remain unclear. Among the many factors related to PD pathology, microglia-mediated neuroinflammation have attracted much attention. Microglia may function in sustaining survival or promoting cell degeneration during PD [19]. Recently, a novel perspective on *α*Syn declares that it is released by dopaminergic neurons and taken up by neighboring cells and describes microglia that can recognize, devour, and digest extracellular *α*Syn [20, 21]. Microglia's response to *α*Syn will therefore be an important determinant of the progression of PD. However, misfolding, oligomerization, and fibrillation of *α*Syn are a cyclic process, and both *α*Syn monomers and aggregates may be secreted by affected neurons via exocytosis [22, 23]. Our study demonstrated that activation of microglia by *α*Syn is based mainly on its aggregation status. PFFs have been conclusively provided with superlative immunogenicity while monomers have similar but weaker immunogenicity and oligomers only exhibited faint immunogenicity. This may partly explain why some patients developed levodopa resistance in late-stage PD. Prion-like PFFs cause a drastic release of proinflammatory cytokines, such as TNF*α* and IL-1*β*, which could promote degeneration of neurons resulting in more leakage of intracellular *α*Syn, finally trapping the process in a vicious spiral. Therefore, interruption of microglial activation has become a promising target against neurodegeneration [19, 24]. Our study further demonstrated that PFFs may be recognized by TLR1/2 and TLR4 and activate NF-*κ*B signaling through IRAK1 and TRAF6. These two key adapter molecules function as a signal transducer which activates IKK in response to proinflammatory cytokines, including TNF*α* and IL-1*β*.

T10 is an active component extracted from the Chinese medical plant, *Tripterygium wilfordii* Hook F, which displays strong anti-inflammatory and immunosuppressive activities. According to previous studies, T10 has been proved to be a negative regulator of the NF-*κ*B pathway in diverse cell models [25, 26]. Consistent with previous studies, T10 exerted a repressive effect on the phosphorylation of NF-*κ*B in the PFF-induced inflammatory model, as well as on the expression of p-IKK*α*/*β*, which is associated with phosphorylation of I*κ*B, an inhibitor of NF-*κ*B. In the TLR/NF-*κ*B pathway, IKK*α*/*β* is a direct target of TRAF6 [6]. In the present study, we did not observe changes in the expression of IRAK1 and TRAF6 following treatment with T10, suggesting that T10 may regulate NF-*κ*B activity via other mechanisms. Additionally, our previous experiments confirmed that T10 did not have a significant effect on the expression of TLR1/2 or TLR4 (data not shown). Besides, PI3K/Akt signaling has also been extensively described as both a positive and a negative regulator of NF-*κ*B activity, which depends mostly on cell type and treatment condition, thus investigated in this study [27]. The results showed that T10 inhibited the expression of both p-PI3K and p-Akt. These findings indicated the following 4 points: (i) PI3K is a positive regulator of NF-*κ*B activity in PFF-treated primary microglia, (ii) PI3K/Akt signaling is upstream of p-IKK*α*/*β* and p-NF-*κ*B, (iii) T10 regulates NF-*κ*B activity through PI3K/Akt pathway, and (iv) IKK*α*/*β* is the downstream component shared by both IRAK1/TRAF6 and PI3K/Akt pathway.

miRNA is a small molecule extensively involved in regulating diverse biological processes, including inflammation as well as development and progression of various human diseases, via the regulation of translational repression, mRNA cleavage, and deadenylation at posttranscriptional stage [28]. Also, miR155-5p has been proven to be related to NF-*κ*B activity following TLR activation [29]. In this study, we showed that stimulation by PFFs leads to the activation of TLR/NF-*κ*B signaling, which, in turn, activates miR155-5p transcription. Furthermore, miR155-5p overexpression serves as an amplifier, which provokes NF-*κ*B activity through the suppression of SHIP1 expression for the robust release of proinflammatory cytokines. Our data shows that miR155-5p provides vital initiation of the inflammatory response to PFFs in primary microglia. T10 halts the flooding of miR155-5p efficiently and stabilizes NF-*κ*B activity within the normal range, avoiding excessive release of proinflammatory cytokines.

SHIP1 is a member of the inositol polyphosphate-5-phosphatase family. It was initially identified as a protein that negatively regulates B-cell receptor signaling. Sly et al. reported that LPS-induced ERK1/2 and Akt phosphorylation as well as cytokines were enhanced in SHIP1^−/−^ BM*φ* cells [30]. Rauh et al. reported that the SHIP1/PI3K axis may manipulate macrophage phenotypes for cancer or inflammation [31]. In the present study, SHIP1 has been identified as a direct target of miR155-5p in primary microglia, verified by the luciferase assay report. Treatment with T10 disrupted the repression of miR155-5p on SHIP1, resulting in reduced phosphorylation of downstream molecules including PI3K, Akt, IKK*α*/*β*, and NF-*κ*B. In order to obtain further insight into the regulatory mechanisms of the miR155-5p/SHIP1 pathway, we analyzed miR155-5p levels and inflammatory cytokine products in T10-treated SHIP1 silenced cells. The results showed that the miR155-5p levels decreased. However, no reductions were observed in the levels of TNF*α* and IL-1*β*, which further demonstrated that SHIP1 is associated with the regulation of T10 on NF-*κ*B activity.

In the present study, T10 has been determined as a potent agent against microglial activation. Recent reports have suggested a role for the T cell infiltration into the substantia nigra in the progression of PD [4, 32]. Furthermore, nitrated-synuclein elicited T cell responses in mice [33]. Whether T10 could inhibit T cell activation and infiltration remains unclear and further studies are felt to be needed.

In conclusion, the current study suggests that PFFs may activate microglia and enhance the release of TNF*α* and IL-1*β* via the TLR/NF-*κ*B pathway. T10 is antagonistic to PFF-mediated inflammation, suppressing NF-*κ*B activity and TNF*α* and IL-1*β* release, by regulating the miR155-5p/SHIP1/NF-*κ*B axis in primary microglia.

## Data Availability

The data used to support the findings of this study are available from the corresponding author upon request.

## Supplementary Materials

Supplementary Materials Supplementary Table 1: primer sequences for quantitative RT-PCR. Supplementary Figure 1: SHIP1 is a direct target of miR155-5p. (a) Predicting results of miR155-5p pairing site in SHIP1 3′UTR based on TargetScan database. There are eight complementary base pairs between mmu-miR-155-5p and SHIP1 3′UTR wild type. (b) Luciferase reporter assay with wild-type SHIP1-3′UTR and mutated SHIP1-3′UTR. The complementary binding site was replaced in the mutant SHIP1-3′UTR. Upregulation of miR155-5p exerts inhibition of luciferase activity in the 3′-UTR-WT group, suggesting that SHIP1 is a direct target of miR155-5p.
