# Supplementary material for: Triptolide Inhibits Preformed Fibril-Induced Microglial Activation by Targeting the MicroRNA155-5p/SHIP1 Pathway
Source: Oxid Med Cell Longev. 2019 Apr 28;2019:6527638. doi: 10.1155/2019/6527638 (PMC6512043; doi:10.1155/2019/6527638)
Supplement: Supplementary Materials — Supplementary Table 1: primer sequences for quantitative RT-PCR. Supplementary Figure 1: SHIP1 is a direct target of miR155-5p. (a) Predicting results of miR155-5p pairing site in SHIP1 3′UTR based on TargetScan database. There are eight complementary base pairs between mmu-miR-155-5p and SHIP1 3′UTR wild type. (b) Luciferase reporter assay with wild-type SHIP1-3′UTR and mutated SHIP1-3′UTR. The complementary binding site was replaced in the mutant SHIP1-3′UTR. Upregulation of miR155-5p exerts inhibition of luciferase activity in the 3′-UTR-WT group, suggesting that SHIP1 is a direct target of miR155-5p. [file 6527638.f1.docx]

**Supplementary materials**

**Supplementary Table 1. Primer sequences for quantitative RT-PCR.**

| Gene | Primer | |
| --- | --- | --- |
| SHIP1 | Forward | 5′- GGCTCCAGCAACCTCCCTCAC-3′ |
|  | Reverse | 5′-TTCTCC GTCTCCACCAAAATCACC-3 |
| GAPDH | Forward | 5’-CTCCTCCACCTTTGACGCTG -3’ |
|  | Reverse | 5’-TCCTCTTGTGCTCTTGCTGG-3’ |
| miR155-5p | Forward | 5’-GCTTCGGTTAATGCTAATCGTG-3 |
|  | Reverse | 5’-CAGAGCAGGGTCCGAGGTA-3’ |
| U6 | Forward | 5’-TGCGGGTGCTCGCTTCGGCAGC-3’ |
|  | Reverse | 5’-CCAGTGCAGGGTCCGAGGT-3’ |

**Supplementary figure 1.**


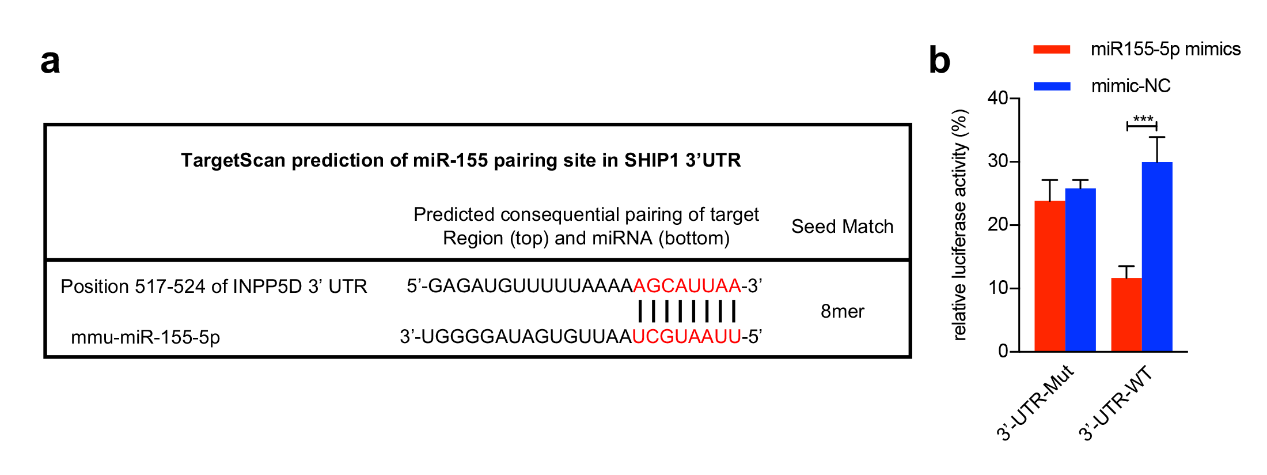


**SHIP1 is a direct target of miR155-5p.** (a) Predicting results of miR155-5p pairing site in SHIP1 3’UTR based on TargetScan database. There are eight complementary base pairs between mmu-miR-155-5p and SHIP1 3’UTR wild type. (b) Luciferase reporter assay with wild-type SHIP1-3’UTR and mutated SHIP1-3’UTR. The complementary binding site was replaced in the mutant SHIP1-3’UTR. Upregulation of miR155-5p exerts inhibition of luciferase activity in the 3’-UTR-WT group, suggesting that SHIP1 is a direct target of miR155-5p.
